# The VvWRKY26-MBW Complex Induced by Salicylic Acid Promotes the Accumulation of Proanthocyanidins in Grape

**DOI:** 10.3390/plants14213272

**Published:** 2025-10-26

**Authors:** Pengfei Zhang, Yuyu Feng, Xiaoran Du, Zhilong Hao, Jinjun Liang, Pengfei Wen

**Affiliations:** College of Horticulture, Shanxi Agricultural University, Jinzhong 030801, China; zpflyl@sxau.edu.cn (P.Z.); 13636721121@163.com (Y.F.); duxiaoran01@163.com (X.D.); 13179934814@163.com (Z.H.); liangjinjun1989@163.com (J.L.)

**Keywords:** vitis, proanthocyanidins, *VvWRKY26*, W-MBW complex, salicylic acid

## Abstract

Proanthocyanidins (PAs) are a significant class of polyphenolic compounds found in grapes, playing important roles in human health and plant stress resistance. Previous studies have shown that the VvMYBPA1/PA2-VvWDR1-VvMYC2 (MBW) complex can regulate the biosynthesis of proanthocyanidins, and some studies have shown that the homologous genes of *VvWRKY26* are involved in the biosynthesis of proanthocyanins and anthocyanins in *Arabidopsis thaliana* and petunias, but the molecular mechanism of *VvWRKY26* in regulating the biosynthesis of proanthocyanins in grapes is still unclear. In this study, we found that the content of proanthocyanidins and the expression of related structural genes were significantly increased by salicylic acid (SA) incubation in grapes during the color transition period. Overexpression of *VvWRKY26* in grapevine healing tissues revealed that overexpression of *VvWRKY26* significantly promoted the accumulation of proanthocyanidins and up-regulation of related structural genes when compared with the empty vector. Further investigation into the interaction mechanisms through yeast two-hybrid and bimolecular fluorescence complementation assays revealed that VvWRKY26 can interact with VvMYBPA1/PA2, VvMYC2, and VvWDR1 to form VvMYBPA1/PA2-VvWDR1-VvMYC2-VvWRKY26 (W-MBW) complex. Through yeast one-hybrid assays and dual-luciferase reporter analysis, it was confirmed that VvWRKY26 could bind to the promoters of VvANR and VvLAR2 and activate their activity. Finally, through the co-overexpression of VvWRKY26 and MBW complex, it was discovered that the promoting activity of *VvANR* and *VvLAR2*, as well as the biosynthesis of PAs, were significantly enhanced, which was much higher than the effect of the MBW complex alone, while the opposite occurred after co-interference. In conclusion, this study explored the role of VvWRKY26 in the biosynthesis of proanthocyanidins in grapes after the interaction with the MBW complex to form W-MBW under SA incubation, providing a new regulatory mechanism for the biosynthesis of proanthocyanidins in grapes.

## 1. Introduction

Proanthocyanidins (PAs) are an important class of polyphenolic compounds in grapes formed by flavan-3-ol monomers and their polymers, which are widely distributed in various tissues in grapes. As an important class of bioactive substances, they have a variety of functions, which are important in human health and plant resistance [1,2].

The synthesis of proanthocyanidins mainly occurs through the phenylpropanoid pathway. First of all, phenylalanine, the assimilation product of plant photosynthesis, is converted into leucoanthocyanidins through the action of phenylalanine lyase (PAL), chalcone synthase (CHS), flavanone hydroxylase (F3H, F3′H or F3′5′H), and dihydroflavonol-4-reductase (DFR), and under the competition of LAR and ANS, leucoanthocyanidins are reduced by LAR to form (+)-catechins; they are also catalyzed by ANS to anthocyanins, which are subsequently converted by ANR to form (−)-epicatechins. (+)-catechins and (−)-epicatechins are the simplest proanthocyanidins in grapes and are the starting units in the condensation process of proanthocyanidins [3]. Two colorless anthocyanin reductases VvLAR1 and VvLAR2 were identified in grapes, and their expressions were quite different, between which VvLAR2 could better respond to adversity.

Previous research indicates that the MYB-bHLH-WD40 protein complex is involved in the biosynthesis of proanthocyanidins. Currently, the research on the MYB family primarily focuses on the R2R3-MYB type [4] which bind to MBS cis-acting elements, including a TAACTG, and regulate structural genes related to proanthocyanidins. bHLH transcription factors influence the accumulation of flavonoids indirectly by forming complexes through interactions with MYB [5]. WD40 protein mainly acts as a stable complex, interacting with MYB and bHLH families through its own WD domain to form MYB-BHLH-WD40 protein complex, which jointly acts on the synthesis pathway of anthocyanins and proanthocyanidins [6]. For example, in *Arabidopsis thaliana*, AtTT2 can interact with TT8 and TTG1 to form the MBW complex, which regulates the biosynthesis of seed coat tannins in Arabidopsis by activating the *AtBAN* promoter [7,8]. In apples, MdMYB9/MdMYB11 forms complexes with MdbHLH3 and MdTTG1, promoting the accumulation of proanthocyanidins by directly binding to the MYB cis-acting elements on the *ANS*, *ANR*, and *LAR2* promoters [9]. The group previously demonstrated that VvMYBPA1/PA2 interacts with VvMYC2 through VvWDR1 to form the VvMYBPA1/PA2-VvWDR1-VvMYC2 complex, which binds to MBS cis-acting elements in the promoters of *VvANR* and *VvLAR1*, thereby promoting the biosynthesis of proanthocyanidins [10,11]. In conclusion, the MYB-bHLH-WD40 protein complex promotes proanthocyanidin accumulation by directly binding to the MYB binding site on the promoters of proanthocyanidin-related structural genes.

Salicylic acid incubation promotes the accumulation of grape phenolics. Yang Bo demonstrated that incubation with 0.5 mmol·L^−1^ salicylic acid promoted the accumulation of phenolics in grape leaves and upregulated the structural genes for flavan-3-ol monomer biosynthesis. Silencing of *VvMYBPA1/PA2* and then incubating with SA revealed that exogenous SA still promoted flavan-3-ol accumulation and up-regulated the expression of *VvMYBPA1/PA2* genes, while increasing the enzymatic activities of ANR and LAR [12]. Zhang Guorong found that the relative expression of the *VvWRKY26* gene was significantly higher than that of the control after the incubation of grape berries by SA osmosis, confirming the involvement of *VvWRKY26* in the salicylic acid signaling pathway and speculating that it may be involved in the biosynthesis of grape proanthocyanidins [13]. The homologous genes of *VvWRKY26*, namely *AtTTG2* and *PhPH3*, are involved in the biosynthesis of proanthocyanidins and anthocyanins in Arabidopsis and petunia [14,15]. PyWRKY26 and PybHLH3 in red pear can combine with each other and act on the promoter of *PyMYB114* together to promote anthocyanin accumulation [16]. Transgenesis of *VvWRKY26* into the petunia ph3 mutant resulted in the restoration of petal color to the wild-type phenotype in the mutant plants [17]. However, it is still unclear how *VvWRKY26* regulates the accumulation of proanthocyanidins in grapes.

In addition, relevant studies have shown that WRKY transcription factors can also regulate the accumulation of proanthocyanidins in plants. McWRKY71 in crabapples can directly bind to the McANR promoter to participate in the regulation of anthocyanins and proanthocyanidins in crabapple, promoting the accumulation of anthocyanins and PAs [18]; CsWRKY31 and CsWRKY48 in tea plants can directly bind to the W-box elements in the promoters of CsLAR, CsDFR, and CsAOMT, inhibiting the biosynthesis of proanthocyanidins [19]. In grapes, VqWRKY56 interacts with VqbZIPC22 to promote the biosynthesis of proanthocyanidins by regulating the expression of VvANR and VvLAR1 promoters [20]—organisms that inhibit proanthocyanidins by down-regulating the expression of VvDFR and VvLAR promoters [21]—and VvWRKY57 binds to the promoter of VvLAR2 to activate its activity and promote the biosynthesis of proanthocyanidins, while the transcriptional activity of VvWRKY20 is inhibited after binding to the promoter of VvLAR2 [22].

Some scholars have also proposed an alternative model for the regulation of flavonoid compounds by transcription factors, that is, WRKY transcription factor combines with MBW complex to form W-MBW complex, which increases the expression of the target genes by enhancing the activity of the MBW complex [23]. Verweij found that *PhPH3*, a homolog of *VvWRKY26*, can act synergistically with the MBW complex to regulate the expression of PhPH1 and PhPH5, affecting vesicle acidification and the accumulation of anthocyanin in *petunia petals* [15]. Amato found that VvWRKY26 could combine with VvMYC1 and VvMYB5a/5b to form the W-MBW complex through VvWDR1 protein, which enhanced the expression of downstream genes *VvCHI*, *VvPH1*, and *VvPH5* and promoted anthocyanin accumulation [17]. Previous studies have shown that salicylic acid incubation can promote the accumulation of proanthocyanidins; additionally, VvMYBPA1/PA2 interacts with VvWDR1 and VvMYC2 to form a ternary complex, which activates the expression of *VvANR* and *VvLAR1*, thereby promoting the biosynthesis of proanthocyanidins [11]. However, it is not clear how salicylic acid promotes proanthocyanidin biosynthesis, so this study takes VvWRKY26 transcription factor as the entry point to further explore its molecular mechanism in SA-induced proanthocyanidin biosynthesis.

## 2. Results

### 2.1. Phylogenetic Analysis of Grape WRKY Family

**Figure 1:**
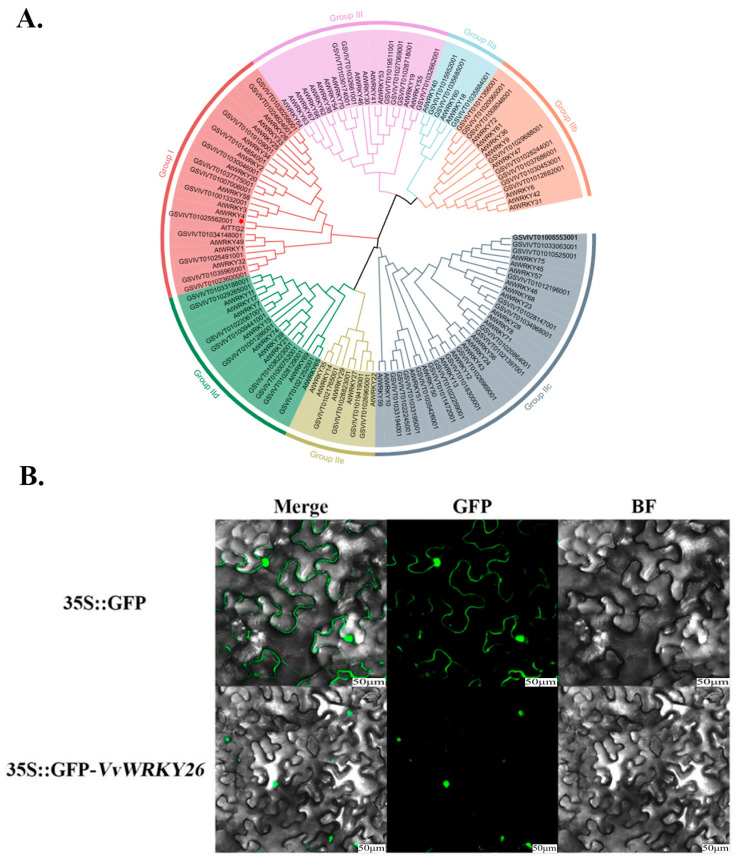
Phylogeny of VvWRKY26. Note: (**A**) Phylogenetic tree of the Arabidopsis and grape WRKY families; (**B**) Subcellular localization of VvWRKY26. Green indicates fluorescence signal.

In order to study the evolutionary relationship, classify the transcription factors of VvWRKY26, and explore its functions, the transcription factors of *Arabidopsis thaliana* and grapevine WRKY family were downloaded from the PlantTFDB database (https://planttfdb.gao-lab.org/index.php (accessed on 22 November 2024)). There are 70 WRKY transcription factors in Arabidopsis and 59 WRKY transcription factors in grapevines. We constructed a neighbor-joining phylogenetic tree for the amino acid sequences of 129 transcription factors. Based on the number of WRKYGQK conserved domains at the N-terminus and the types of zinc finger structure in the WRKY transcription factor amino acid sequences, this family of transcription factors was divided into three major groups, with Group II further subdivided into five subgroups. Group I transcription factors have two WRKY conserved domains and a C2H2-type zinc finger structure, and both Group II and Group III transcription factors have only one WRKY conserved domain, with Group II having the same zinc finger structure type as Class I, while Group III has a C2HC zinc finger structure type. From the phylogenetic tree, we can find that the transcription factor VvWRKY26 (GSVIVT01025562001) belongs to the group I of transcription factors in the WRKY family (Figure 1A) and is in the same class as AtTTG2. Moreover, AtTTG2 has been reported to be involved in the biosynthesis of flavonoids in *Arabidopsis thaliana*. Therefore, it is speculated that VvWRKY26 also has similar biological functions.

In order to verify the function of the VvWRKY26 transcription factor, a recombinant vector 35S::GFP-*VvWRKY26* was constructed for subcellular localization using an empty vector as a control. Through laser confocal microscopy, it was found that the green, fluorescent signal of VvWRKY26 was located solely in the nucleus, while the green, fluorescent signal of the empty vector was dispersed throughout the entire cell, indicating that VvWRKY26 is localized in the nucleus (Figure 1B).

### 2.2. The Incubation of SA Promotes the Accumulation of VvWRKY26 in Grapes

**Figure 2:**
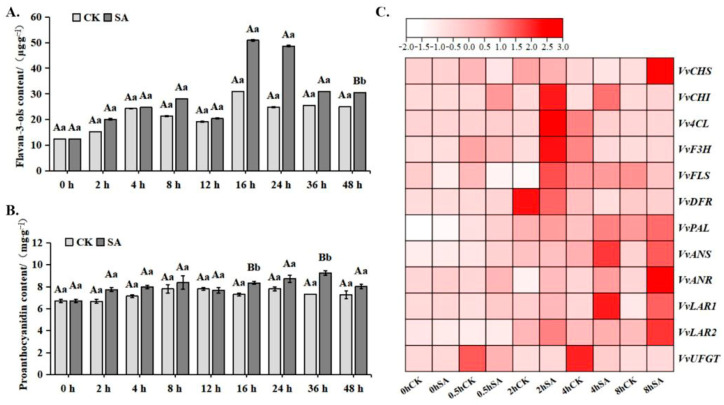
Effect of SA treatment on flavanetriol and proanthocyanidins. Note: (**A**) Content of flavan-3-ols after SA treatment (μg·g^−1^). (**B**) Content of proanthocyanidins after SA treatment (mg·g^−1^). (**C**) Expression of structural genes related to flavonoid metabolism after SA treatment. Different lowercase letters indicate significant differences (*p* < 0.05); different capital letters indicate extremely significant differences (*p* < 0.01).

To further investigate how *VvWRKY26* regulates the biosynthesis of proanthocyanidins under salicylic acid induction, we employed the method of SA infiltration to incubate grape berries. By measuring the content of proanthocyanidins and flavan-3-ols in the berries, we found that the proanthocyanidin content in the grape berries increased after SA infiltration incubation, and starting from 2 h post-treatment, the proanthocyanidin content was significantly higher than that of the control group (Figure 2A), and the average content of flavan-3-ols was also higher than that of the control (Figure 2B). Furthermore, by measuring the relative expression levels of flavonoid biosynthesis structural genes in grape berries, we found that the expression levels of *VvCHI*, *VvPAL*, *VvANR*, and *VvLAR1* were higher in the SA-treated group compared to the control group within 0.5 to 8 h, while the relative expression levels of *VvANR* and *VvLAR2* began to be upregulated starting at 0.5 h and 2 h, respectively, both of which were also higher than those of the control group (Figure 2C).

### 2.3. Overexpression of VvWRKY26 Promotes the Accumulation of Proanthocyanins in Grape Callus

**Figure 3:**
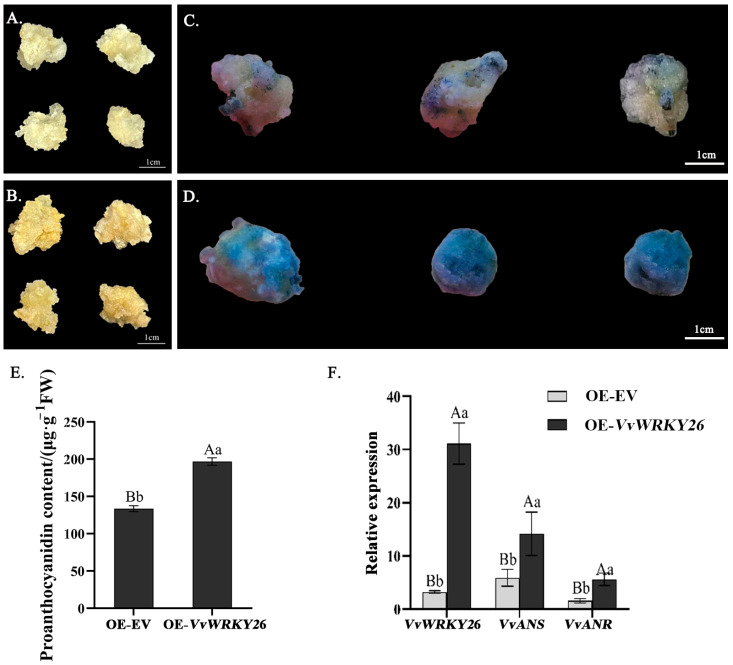
*VvWRKY26* overexpressing in healing grapevine tissue. Note: (**A**) Phenotypic observation of healing null tissues. (**B**) DMACA staining of healing null tissues. (**C**) Phenotypic observation of *VvWRKY26* overexpression in healing tissues. (**D**) DMACA staining of *VvWRKY26* overexpression in healing tissues. (**E**) Proanthocyanidin content of healing tissues in overexpressed—the empty vector (OE-EV)—and overexpressed *VvWRKY26* (OE-*VvWRKY26*) (μg·g^−1^ FW). (**F**) Relative expression of genes. Different lowercase letters indicate significant differences (*p* < 0.05); different capital letters indicate extremely significant differences (*p* < 0.01).

In order to verify the role of *VvWRKY26* in the biosynthesis of proanthocyanidins in grapes, 20 mg/L hygromycin was used for callus resistance screening. After approximately 4 weeks of cultivation, it was observed that the OE-*VvWRKY26* callus exhibited a darker color compared to the empty vector control (Figure 3A,C). After DMACA staining, the healing tissues of all experimental groups were found to show distinct deep blue coloration, whereas the control group appeared to have a small amount of blue color (Figure 3B,D), indicating that overexpression of *VvWRKY26* can promote the accumulation of proanthocyanidins in healing grapevine tissues (Figure 3E). The relative expression levels of *VvWRKY26*, *VvANS*, and *VvANR* genes in the healing tissues of different treatments was detected by qRT-PCR (Figure 3F), the results showed that the relative expression of *VvWRKY26*, *VvANS*, and *VvANR* genes in the healing tissues overexpressing *VvWRKY26* was significantly higher than that in the empty vector control, which further indicated that overexpression of *VvWRKY26* could promote the accumulation of proanthocyanidins and activate the expression of related genes in the healing grapevine tissues.

### 2.4. VvWRKY26 Interacts with the MBW Complex to Form a Tetramer

To further investigate whether VvWRKY26 regulates proanthocyanidin biosynthesis through the MBW complex, the corresponding vectors were constructed for yeast two-hybrid and bimolecular fluorescence complementation assays.

**Figure 4:**
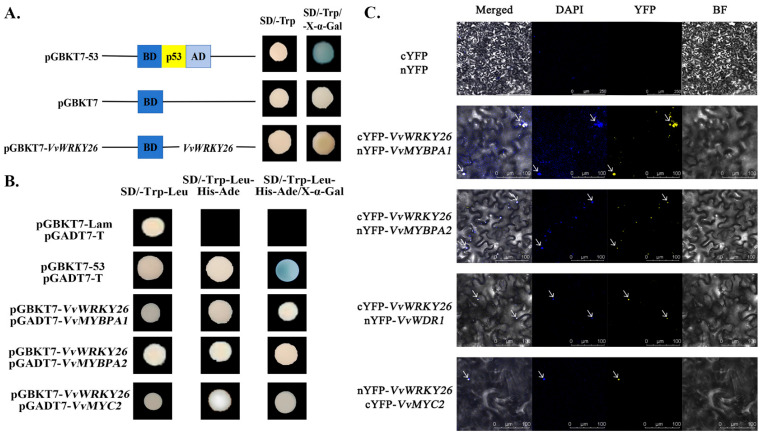
VvWRKY26 interacts with the MBW proteins to form tetramers. Note: (**A**) Transcriptional self-activating activity analysis. (**B**) Yeast two-hybrid results. (**C**) Bimolecular fluorescence complementation results. The position indicated by the white arrow in the figure is where the fluorescence signal appears.

The results of the transcription–self-activation experiment are shown in Figure 4A. On the SD/-Trp/X-α-Gal plate, the positive control turned blue, while the negative control and pGBKT7-VvWRKY26 did not turn blue, indicating that the protein encoded by VvWRKY26 does not exhibit yeast self-activation. The yeast two-hybrid results are shown in Figure 4B. The positive group, pGBKT7-*VvWRKY26* and pGADT7-*VvMYBPA1*, pGBKT7-*VvWRKY26* and pGADT7-*VvMYBPA2*, pGBKT7-*VvWRKY26* and pGADT7-*VvMYC2*, co-transformed yeast that could grow on the SD/-Trp-Leu-His-Ade quadruple dropout medium plates. When grown on SD/-Trp-Leu-His-Ade/X-α-Gal plates, the positive control group turned blue, but none of the experimental groups turned blue, indicating that VvWRKY26 could interact with VvMYBPA1, VvMYBPA2, and VvMYC2, but the interaction effect was weak. The results of the bimolecular fluorescence complementation assay showed that *VvWRKY26* could produce yellow fluorescence signals in the nucleus after co-transformation with *VvMYBPA1*, *VvMYBPA2*, *VvMYC2*, and *VvWDR1* into tobacco leaves, whereas the control group of cYFP and nYFP were unable to produce yellow fluorescence signals (Figure 4C). This indicates that VvWRKY26 can interact with VvMYBPA1,VvMYBPA2, VvMYC2, and VvWDR1.

In conclusion, both in vivo and in vitro assays confirmed that VvWRKY26 can interact with VvMYBPA1/PA2, VvMYC2, and VvWDR1 to form the W-MBW complexes, which can further promote the biosynthesis of proanthocyanidins.

### 2.5. The Overexpression of the W-MBW Complex Promotes the Biosynthesis of Proanthocyanidins in Grape Leaves and Berries

**Figure 5:**
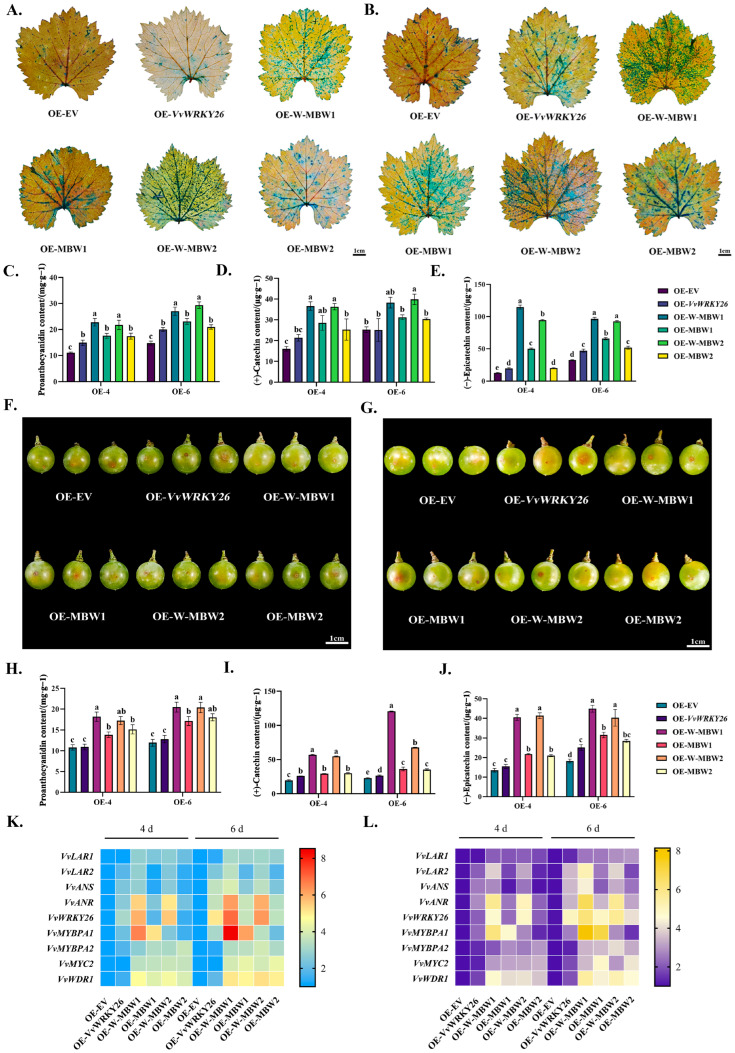
Effect of overexpression of W-MBW complex on grape proanthocyanidins. Note: (**A**) Results of DMACA staining of leaves at 4 d. (**B**) Results of DMACA staining of leaves at 6 d. (**C**) Content of proanthocyanidins in leaves (mg·g^−1^). (**D**) Content of catechin monomersin leaves (μg·g^−1^). (**E**) Content of epicatechin monomers in leaves (μg·g^−1^). (**F**) At 4 days post-injection in berries. (**G**) At 6 days post-injection in berries. (**H**) Content of proanthocyanidins in berries (mg·g^−1^). (**I**) Content of catechin monomers in berries (μg·g^−1^). (**J**) Content of epicatechin monomers in berries (μg·g^−1^). (**K**) Expression of genes related to proanthocyanidin biosynthesis in leaves. (**L**) Expression of genes related to proanthocyanidin biosynthesis in berries. Different lowercase letters indicate significant differences (*p* < 0.05).

In order to further investigate the effect of VvWRKY26 and the MBW complex on proanthocyanidin biosynthesis, transient overexpression in leaves was performed via vacuum infiltration. Samples were taken after 4 days and 6 days of culturing, followed by DMACA staining; blue coloration indicated proanthocyanidin content. It was found that in the presence of the W-MBW complex, the area proportion of blue was higher than that of the MBW complex, and significantly higher than that of *VvWRKY26* alone (Figure 5A,B). In addition, by measuring the content of proanthocyanidins in the leaves using a spectrophotometer, the results indicate that compared to the control group, the highest proanthocyanidin content was observed when VvWRKY26 and the MBW complex were co-overexpressed, followed by the MBW complex, while the lowest content was observed when *VvWRKY26* was overexpressed alone (Figure 5C). The content of flavan-3-ol monomers under different treatments was determined by HPLC; the results showed that compared to the control group, when the MBW1 trimer was overexpressed, the catechin content increased from 25.26 μg·g^−1^ to 35.47 μg·g^−1^ after 6 days, and the epicatechin content increased from 32.46 μg·g^−1^ to 65.68 μg·g^−1^. When the MBW2 trimer was overexpressed, the catechin content increased to 30.40 μg·g^−1^, and the epicatechin content increased to 51.67 μg·g^−1^; however, when *VvWRKY26* and the MBW complex were co-overexpressed, the contents of catechin and epicatechin were significantly higher than those in the other treatment groups (Figure 5D,E). The expression levels of related genes were quantified by quantitative fluorescence, and the gene heatmap revealed that compared to the control, the expression of the structural genes related to the biosynthesis of proanthocyanidin—including *VvLAR1*, *VvLAR2*, *VvANR*, and *VvANS*—were upregulated during the transient overexpression of the W-MBW complex. Among these, *VvANR* showed a highly significant upregulation (Figure 5K).

In addition to leaves, we also conducted studies in grape berries using a syringe injection method (Figure 5F,G). As above, we determined the content of proanthocyanidins and flavan-3-ol monomers in the berries with the butanol-hydrochloric acid method and high-performance liquid chromatography (HPLC), and performed qRT-PCR for the related genes. The results indicate that when the MBW1 complex was overexpressed, the proanthocyanidin content increased to 1.47 times compared to the control, and when the MBW2 complex was overexpressed, the total content of proanthocyanidins increased to 1.51 times. When *VvWRKY26* was co-overexpressed with the MBW complex, the proanthocyanidin content increased to 1.72 times and 1.71 times, respectively (Figure 5H). By analyzing the changes in the content of flavan-3-ol monomers under different treatments, it could be observed that when *VvWRKY26* was co-expressed with the MBW complex, the contents of catechin and epicatechin were significantly higher than those of other treatment groups. At the sampling on day 4, compared to the control, the content of catechin had significantly increased by 2.93 times after the overexpression of the W-MBW1, while the content of epicatechin had significantly increased by 3.00 times; after the overexpression of the W-MBW2, the catechin content had increased by 2.81 times, and the epicatechin content had increased by 3.10 times (Figure 5I,J). The expression of the related genes was detected by qRT-PCR, and the results of the relevant expression of each gene were basically the same as those in the overexpressed leaves. When the four transcription factors were co-expressed, the structural genes involved in proanthocyanidin biosynthesis, *VvLAR1*, *VvLAR2*, *VvANR*, and *VvANS*, were all upregulated, and the up-regulation ratio was significantly higher than that in the other treatment groups (Figure 5L).

### 2.6. The RNA Interference of the W-MBW Complex Inhibits Proanthocyanidin Content in Grape Leaves and Berries

**Figure 6:**
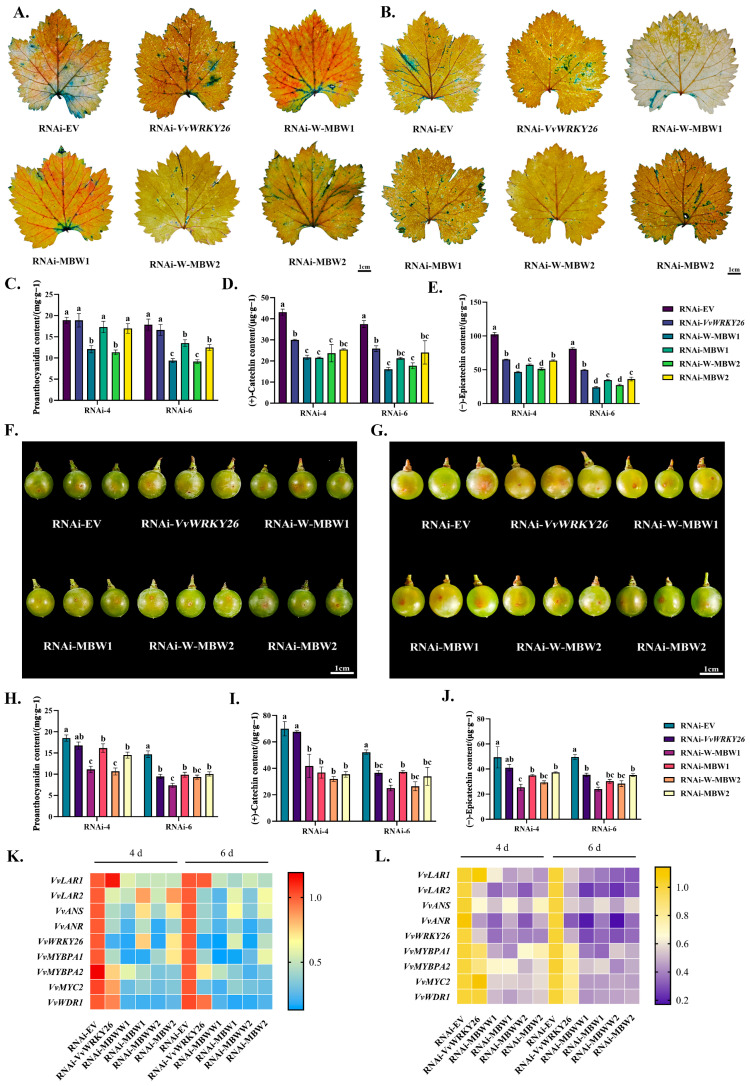
Effect of RNAi-W-MBW complex on grape proanthocyanidins. Note: (**A**) Results of DMACA staining of leaves at 4 d. (**B**) Results of DMACA staining of leaves at 6 d. (**C**) Content of proanthocyanidins in leaves (mg·g^−1^). (**D**) Content of catechin monomers in leaves (μg·g^−1^). (**E**) Content of epicatechin monomers in leaves (μg·g^−1^). (**F**) At 4 days post-injection in berries. (**G**) At 6 days post-injection in berries. (**H**) Content of proanthocyanidins in berries (mg·g^−1^). (**I**) Content of catechin monomers in berries (μg·g^−1^). (**J**) Content of epicatechin monomers in berries (μg·g^−1^). (**K**) Expression of genes related to proanthocyanidin biosynthesis in leaves. (**L**) Expression of genes related to proanthocyanidin biosynthesis in berries. Different lowercase letters indicate significant differences (*p* < 0.05).

We constructed the recombinant vectors *VvWRKY26*-RNAi, *VvMYBPA1*-RNAi, *VvMYBPA2*-RNAi, *VvMYC2*-RNAi, and *VvWDR1*-RNAi. After mixing the different interference vector suspensions in a 1:1 ratio and performing the vacuum infiltration of the grape leaves, it could be observed through DMACA staining that the control group exhibited the largest blue area ratio, followed by the leaves, after *VvWRKY26* interference alone, while the blue area ratio was the smallest when all four transcription factors were co-interfered (Figure 6A,B). Further determination of the content of proanthocyanidin and flavan-3-ol in the leaves revealed that the changes in the content of these substances were consistent with the leaf phenotype. Compared with the control group, the content of proanthocyanidins decreased from 17.87 mg·g^−1^ to 9.39 mg·g^−1^ at 6 days after simultaneous interfering from *VvWRKY26*, *VvMYBPA1*, *VvMYC2*, and *VvWDR1* (Figure 6C), the catechin content decreased from 37.47 μg·g^−1^ to 16.12 μg·g^−1^ (Figure 6D), and the epicatechin content decreased from 81.14 μg·g^−1^ to 23.88 μg·g^−1^ (Figure 6E). When *VvWRKY26*, *VvMYBPA2*, *VvMYC2*, and *VvWDR1* were co-interfered, the changes in proanthocyanidin and flavan-3-ol monomer content were almost the same as those after interference with W-MBW1, and the inhibition of proanthocyanidin biosynthesis was the most significant. These results were the opposite of those observed after the overexpression of the W-MBW complex. RNA was extracted from leaves of different treatment groups, reverse transcribed to cDNA, and then used as a template to determine the relative expression of related genes; the results showed that only *VvWRKY26* expression was significantly down-regulated when *VvWRKY26* was interfered with, and at the same time, the relative expression of the proanthocyanidin biosynthesis structural genes *VvLAR2*, *VvANR*, and *VvANS* were reduced, while the change in *VvLAR1* was insignificant, and the expression of *VvMYBPA1/PA2*, *VvMYC2*, and *VvWDR1* was significantly reduced. With the MBW trimer interfering, the expression of *VvLAR1* and *VvANR* was significantly reduced, the relative expression of *VvANS* was reduced but not significantly, and the relative expression of *VvLAR2* was insignificantly changed, but when the four transcription factors were co-interfered, the relative expression of *VvLAR1*, *VvLAR2*, *VvANS* were significantly reduced and *VvANR* was highly significantly downregulated (Figure 6K).

Different interference recombinant vector plasmids were introduced into Agrobacterium and injected into grape berries (Figure 6F,G), measuring the contents of different treatment groups of proanthocyanidins and flavan-3-ol monomers and determining the relative expression levels of related genes. The results indicated that when the four transcription factors were simultaneously interfered with, the proanthocyanidin content decreased the most, at 0.58 times that of the control group. (Figure 6H). The contents of catechin and epicatechin monomers were also significantly lower than those in the other treatment groups, with catechin reduced by approximately 0.47 times and epicatechin reduced by approximately 0.46 times compared to the control group (Figure 6I,J). The expression levels of related transcription factors and structural genes involved in proanthocyanidin biosynthesis were also at their lowest levels, which was exactly the opposite of the results of overexpressing grape berries (Figure 6L).

Based on the results of the above experiments, it can be concluded that when *VvWRKY26* is co-overexpressed with the MBW complex, the structural genes involved in the biosynthesis of proanthocyanidin are significantly upregulated, leading to the greatest change in proanthocyanidin biosynthesis; followed by the overexpression of the MBW complex alone; when *VvWRKY26* is overexpressed alone, it also promotes the biosynthesis of proanthocyanidins, but not significantly as in the other treatment groups.

### 2.7. VvWRKY26 Regulates VvANR and VvLAR2 Through the MBW Complex

**Figure 7:**
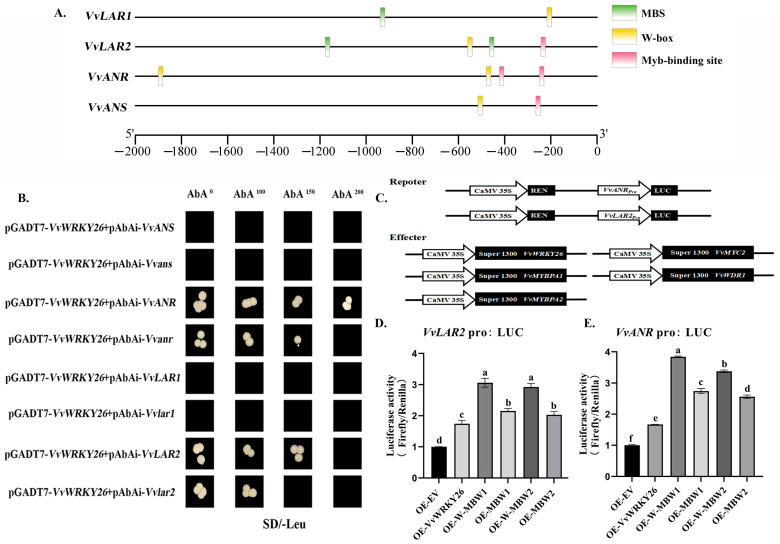
Effect of W-MBW on *VvANR*, *VvLAR2* promoter. Note: (**A**) Analysis of promoter cis-acting elements of *VvANR*, *VvANS*, *VvLAR1*, and *VvLAR2*; (**B**) Y1H validation of VvWRKY26-promoter interaction; (**C**) Schematic diagram of dual-luciferase vectors; (**D**) Effect of W-MBW on the promoter activity of *VvLAR2* gene; (**E**) Effect of W-MBW on the promoter activity of *VvANR* gene. Different lowercase letters indicate significant differences (*p* < 0.05).

To investigate whether *VvWRKY26* can directly regulate downstream structural genes for proanthocyanidins biosynthesis, we analyzed promoter cis-acting elements and performed yeast one-hybrid and dual-luciferase assays. The results showed that the structural genes *VvLAR1*, *VvLAR2*, and *VvANS* all contain one W-box on the 2000 bp sequence of the promoter upstream of the CDS region, while *VvANR* has two W-boxes (TTGACC) (Figure 7A).

Short fragments containing the W-box nearest to the ATG in the promoters of *VvLAR1*, *VvLAR2*, *VvANR*, and *VvANS* were selected to construct pBait-AbAi vectors for yeast one-hybrid assay; the W-boxes were located at positions −206, −550, −469, and −506. The results showed that after *VvWRKY26* was co-transformed with four structural genes regulating the biosynthesis of proanthocyanidins, pGADT7-*VvWRKY26*+pAbAi-*VvANR* and pGADT7-*VvWRKY26*+pAbAi-*VvLAR2* could be grown normally on SD/-Leu/AbA medium, while the negative control group, pGADT7-*VvWRKY26*+pAbAi-*VvANS* and pGADT7-*VvWRKY26*+pAbAi-*VvLAR1* could not grow normally on SD/-Leu medium, suggesting that VvWRKY26 can directly bind to the W-box of *VvANR* and *VvLAR2* promoters (Figure 7B).

Meanwhile, reporter vectors and effector vectors were constructed for dual-luciferase assay, and the vector construction method illustrated in the figure (Figure 7C). The results showed that when *VvWRKY26* was overexpressed, the promoter activities of *VvANR* and *VvLAR2* were significantly enhanced, with the former increased by about 67% and the latter increased by about 74% (Figure 7D,E). Y1H and LUC assays confirmed that *VvWRKY26* could directly bind to and enhance the promoter activities of *VvANR* and *VvLAR2*, thereby regulating the biosynthesis of proanthocyanidins.

To further investigate how *VvANR* and *VvLAR2* are regulated by the complex, corresponding vectors were constructed for dual-luciferase assays. By measuring the activity of luciferase, it was found that the co-overexpressed of *VvWRKY26*, *VvMYBPA1/PA2*, *VvMYC2*, and *VvWDR1* proteins significantly promoted the promoter activities of *VvANR* and *VvLAR2*. When only three transcription factors *VvMYBPA1/PA2*, *VvMYC2* and *VvWDR1* were co-overexpressed with the promoter reporters, the LUC activities of pro*VvANR* and pro*VvLAR2* were lower than when all four transcription factors were present (Figure 7D,E). These results indicate that VvWRKY26 can enhance the ability of the MBW complex to activate *VvANR* and *VvLAR2*.

## 3. Discussion

### 3.1. Phylogenetic Analysis of the WRKY Family in Grapevine

The WRKY family of Group I transcription factors involved in the biosynthesis of flavonoid compounds. Previous studies on grapevine WRKY transcription factors identified 59 transcription factors with distinct WRKY domains from the grapevine whole-genome database, of which VvWRKY26 belongs to group I [24], which is consistent with the results of this study. Currently, there are also many reports regarding the involvement of group I WRKY transcription factors in flavonoid biosynthesis. In *Arabidopsis thaliana*, *AtTTG2* promotes the biosynthesis of proanthocyanidins by positively regulating *TT12* and *TT13* on the vacuolar membrane [25]. In pears, PyWRKY26 interacts with PybHLH3 to form a complex that targets the *PyMYB114* promoter, thereby influencing anthocyanin accumulation [16]. Ahmed Alabd found that PpWRKY44 could positively regulate anthocyanin biosynthesis through the transcriptional regulation of *PpMYB10*, significantly enhancing the accumulation of this compound [26]. In grapevines, *VvWRKY26* enhances flavonoid biosynthesis through *VvCHI* [24]. This study indicates that VvWRKY26 is a Group I transcription factor of the grape WRKY family, clustered in the same branch as AtTTG2, and it is speculated that they have similar molecular biological functions. Further research has found that VvWRKY26 plays a positive regulatory role in the biosynthesis of proanthocyanidins, which is consistent with the results of previous studies.

### 3.2. VvWRKY26 Positively Regulates SA-Mediated Proanthocyanidin Biosynthesis in Grapevines

SA treatment can increase the content of phenolic compounds in grapevines. A previous study showed that the contents of total phenols, total flavonoids, total flavan-3-ols, and flavan-3-ol monomers in grape berries were significantly increased and higher than the control after exogenous application of SA [27]. Salicylic acid and jasmonic acid induced enhanced production of total phenolics, flavonoids, and antioxidant metabolism in callus cultures of *Givotia moluccana* (L.) Sreem [28]. The results of Yang Bo’s experiment showed that the contents of total phenols, total flavonoids, total flavan-3-ols, and flavan-3-ol monomers in grape leaves could be increased after exogenous SA treatment [29] The expressions of *VvANR*, *VvLAR1*, *VvLAR2*, and *VvMYBPA1* were significantly up-regulated after 1 h of incubation, and after 3 h of incubation, the expressions of *VvANR*, *VvLAR2*, *VvMYBPA1*, and *VvMYBPA2* were significantly higher than those of the control group, and there was no significant difference in other genes, indicating that *VvLAR2* could better respond to the defense mechanism triggered by salicylic acid. When *VvMYBPA1* and *VvMYBPA2* were silenced and then incubated with SA, it was found that exogenous SA could promote the gene expression of *VvMYBPA1/PA2* and the enzymatic activities of ANR and LAR, enhancing the accumulation of flavan-3-ol monomers [30]. It has also been shown that VvLAR2 expression is significantly induced under drought conditions, whereas the expression of *VvLAR1* is mainly regulated by the stage of fruit development. Zhang Guorong’s experimental results indicated that the expression level of *VvWRKY26* after SA treatment was significantly higher than that of the control [13]. Our results showed that after 150 μmol/L SA vacuum infiltration and incubation in grape berries at the veraison stage, the biosynthesis of flavonoid compounds was promoted and the expression of *VvANR*, *VvLAR1*, and *VvLAR2* was up-regulated, which was basically similar to the previous studies, confirming that *VvWRKY26* participated in SA-induced proanthocyanidin biosynthesis through up-regulation of the structural gene expression.

### 3.3. The VvWRKY26-MBW Complex Regulates the Biosynthesis of Proanthocyanidins in Grapevine

Previous studies have shown that after *VvWRKY26* transgenic infiltration, petunia *ph3* mutants can restore their petal colors from light pink to deep red, indicating that this transcription factor can promote the accumulation of anthocyanin in petals of petunias through heterologous expression [17]. At the same time, it has been reported that the content of flavonols and proanthocyanidins in the seed coats of black-seeded rapeseed is higher than in yellow-seeded rapeseed [31], and that *BnTTG2*, as a homologous gene of *VvWRKY26* in rapeseed, is differentially expressed in the accumulation of flavonoid compounds in the seed coats of black-seeded and yellow-seeded rapeseed, with higher expression in black-seeded rapeseed than in yellow-seeded rapeseed during most developmental stages, indicating that *BnTTG2* can promote the biosynthesis of flavonoid compounds [32]. In this study, in order to investigate the fundamental functions of *VvWRKY26*, the leaves and berries were infected by this transcription factor by Agrobacterium at the veraison stage, as were grapevine healing tissues, using overexpression and interference techniques, to determine the content of proanthocyanidins and the relative expression of their biosynthetic structural genes, and it was found that the content of proanthocyanidins in the overexpressed samples was significantly higher than that in the control group, and the relative expression of the structural genes was also significantly higher than in the control group. The relative expression of the structural genes was also significantly higher than that of the control, indicating that *VvWRKY26* could promote the accumulation of proanthocyanidins, which was similar to the results of previous studies.

The molecular mechanisms by which transcription factors regulate the biosynthesis of plant secondary metabolites are complex and diverse, they can not only directly bind to the cis-acting elements of downstream structural genes to regulate the expression of target genes, but also indirectly regulate the expression of target genes by forming complexes through interactions with other transcription factors. In apples, MdWRKY41 can negatively regulate the biosynthesis of proanthocyanidins and anthocyanidins by interacting with MdMYB16 to inhibit the expression of downstream *VvANR* and *VvUFGT* [5]. MdMYB9 and MdMYB11 can form MBW complexes with MdbHLH3 and MdTTG1, which regulate the accumulation of proanthocyanidins by binding to the promoters of structural genes [9]. A previous study by our group showed that VvMYBPA1/PA2 can interact with VvWDR1 and VvMYC2 to form an MBW trimer, regulating the downstream structural genes *VvLAR1* and *VvANR*, thereby promoting the biosynthesis of proanthocyanidins [11]. In this study, the results of bimolecular fluorescence complementation and yeast two-hybrid assays showed that VvWRKY26 could interact with VvMYBPA1/PA2, VvMYC2, and VvWDR1 to form a protein complex, confirming that VvWRKY26 can form a W-MBW protein complex with the MBW trimer and regulate the expression of the downstream structural genes. This conclusion is similar to previous research on the function of *PhPH3*, a homologous gene of *VvWRKY26* in petunias, where the PH4-AN1-AN11 MBW complex in petunias can regulate the expression of *PhPH3*, and PhPH3 can interact with the WD40 protein AN11, indicating that the WRKY transcription factor PhPH3 targets the expression of downstream structural genes *PH1* and *PH5* by joining the MBW complex, thereby promoting the biosynthesis of anthocyanins and changing the color of petunia petals [15].

In this study, VvWRKY26 interacted with MBW trimer to form the W-MBW protein complex, targeting the expression of downstream *VvANR* and *VvLAR2*, and thus regulating the biosynthesis of proanthocyanidins, but did not directly bind to *VvANS* and *VvLAR1*. By analyzing the cis-acting elements on the promoters of *VvANR*, *VvANS*, *VvLAR1*, and *VvLAR2*, it was found that in the promoters of *VvANR* and *VvLAR2*, the MYB and WRKY family-transcription-factor-specific binding sites, MBS and W-box, were located close to each other. The W-box was located at −469, and the Myb-binding site at position −239, on the *VvANR* promoter. On the *VvLAR2* promoter, the W-box was located at −550 and the MBS at position −456. It was speculated that due to the proximity of these cis-acting elements, VvWRKY26 interacted with the MBW trimer to form the W-MBW protein complex, which jointly acted on the promoters of these two structural genes, participating in the biosynthesis of proanthocyanidins.

It has been reported that the WRKY transcription factors interact with the MBW complex to form the W-MBW complex, which can enhance the activation of downstream structural genes. VvWRKY26 forms a tetramer with MYB5a/5b+MYC1+WDR1, regulating the expression of VvCHI, and the addition of VvWRKY26 can improve the ability of MYB5a/5b to activate downstream target genes by dual-luciferase assay [17]. In this study, by comparing the differences in the content of proanthocyanidins, catechins, and epicatechins and the relative gene expression levels and luciferase activity between the MBW complex and the W-MBW complex treatments, it was found that the promotion of proanthocyanidin biosynthesis was significantly stronger with the W-MBW complex than with the MBW complex, which suggests that the addition of VvWRKY26 also enhances the effect of the MBW complex on proanthocyanidin biosynthesis, which is similar to the findings of previous research.

**Figure 8:**
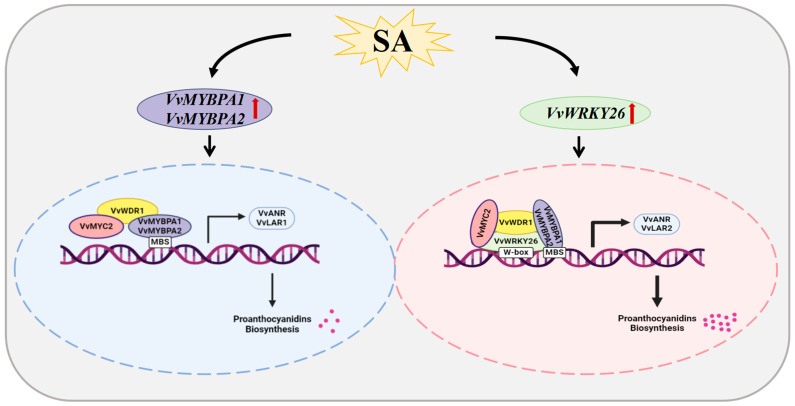
Model diagram of SA-induced regulation of proanthocyanidins biosynthesis in grapevine by the VvWRKY26-MBW complex.

Based on the comprehensive experimental results, we concluded that VvWRKY26 responds to SA induction and promotes the biosynthesis of proanthocyanidins by interacting with VvMYBPA1/PA2, VvMYC2, and VvWDR1 to form the W-MBW tetramer, up-regulating the expression of the structural genes *VvANR* and *VvLAR2*, thereby promoting proanthocyanidin biosynthesis (Figure 8).

## 4. Materials and Methods

### 4.1. Plant Materials

Grapes (*Vitis vinifera* L. cv. ‘Zaoheibao’) were planted in the greenhouse of the horticulture station of Shanxi Agricultural University and cultivated on a hedge, with a row spacing of 1.0 m × 2.5 m, and were managed under conventional cultivation. Fresh shoot leaves and grapes in the early stage of veraison were collected as vacuum infiltration and injection materials, and the samples were immediately frozen in liquid nitrogen and stored in a −80 °C refrigerator for later use.

*Nicotiana benthamiana* was grown in a light incubator under the following conditions: temperature 25 °C; humidity 70%; light intensity 10,000 lx; 16 h/8 h alternating light and dark, and conventional water and fertilizer management.

### 4.2. Bioinformatics Analysis

The WRKY family transcription factors of Arabidopsis and grape were downloaded from the PlantTFDB database (https://planttfdb.gao-lab.org/index.php (accessed on 22 November 2024)). The MEGA 11 was utilized to construct a phylogenetic tree, which was subsequently refined and visualized using the Evolview website (https://www.evolgenius.info/evolview/#/login (accessed on 24 November 2024)).

Download the promoter sequence of 2000 bp upstream of the relevant gene ATG from the NCBI database (https://www.ncbi.nlm.nih.gov/ (accessed on 14 October 2023)) and import it into the promoter visualization website PlantCARE (https://bioinformatics.psb.ugent.be/webtools/plantcare/html/ (accessed on 23 November 2023)) to analyze the cis-acting elements.

### 4.3. RNA Extraction and Real-Time Fluorescent Quantitative PCR

Total RNA from plant tissues was extracted using the CTAB method (with isopropanol precipitation), purified using an RNA purification kit (BestEnzymes. Lianyungang, JiangSu. China) and then stored at −80 °C for future use. The reverse transcription kit (BestEnzymes. Lianyungang, JiangSu. China) was used to convert it into cDNA, it was stored at −20 °C, and the concentration was adjusted as needed for subsequent experiments.

Fluorescence quantification primers for relevant genes were designed by Primer Premier 5. The concentration of reverse transcribed cDNA was diluted to 200 ng/μL as a template with *VvUBQ* used as the reference gene, the reaction system was 20 μL, and the real-time PCR procedure was referenced to the 2 × Realtime PCR Super mix, in which the annealing temperature was 58.2 °C. After the reaction, the relative gene expression levels were calculated using the 2^−△△Ct^ method. The Primer sequences used in Table A1.

### 4.4. Salicylic Acid Incubation of Grapes

The grapes were incubated in 150 μmol/L SA medium (50 mmol/L MES, 5 mmol/L CaCl_2_, 5 mmol/L MgCl_2_, 1 mmol/L EDTA, 5 mmo/L ascorbic acid, pH 5.5), while the control group was placed in the incubation medium without SA (incubation medium volume:fruit volume = 3: 1). Samples underwent vacuum osmotic treatment for 20 min (vacuum degree is 0.1 MPa), and stood for 30 min after decompression. After removing the incubation medium, the fruits were rinsed three times with ddH2O, wrapped in wet gauze, and stored in a constant temperature incubator at 28 °C (±1 °C) in the dark. Samples were taken at 0 h, 0.5 h, 2 h, 6 h, 8 h, 12 h, 16 h, 24 h, 36 h, and 48 h. The fruits were frozen in liquid nitrogen and stored at −80 °C.

### 4.5. Subcellular Localization

The full-length CDS of *VvWRKY26* without the stop codon was inserted into the pCAMBIA-1300-35S-GFP vector using T4 DNA ligase to obtain the recombinant vector 35S: GFP-*VvWRKY26*.

The recombinant vector plasmid and the control empty vector plasmid were transformed into Agrobacterium GV3101, after resuspension, and the bacteria was injected into the abaxial side of leaves from four-week-old tobacco plants. After being placed in a light incubator for 12 h of dark and then cultured alternately in light and dark cycles for 2 days, the fluorescence signals were observed using a confocal laser scanning microscope. The Primer sequences used in Table A1.

### 4.6. Bimolecular Fluorescence Complementation

The full-length sequence of *VvWRKY26*, lacking the stop codon, was double-digested and then inserted into the pCAMBIA-1300-35S-YFPc and pCAMBIA-1300-35S-YFPn vectors using T4 DNA ligase to construct bimolecular fluorescence complementation vectors.

The recombinant plasmids and the empty vector plasmids were transformed into *Agrobacterium tumefaciens* GV3101. The different combinations of infection solution were mixed in a 1:1 ratio and then infiltrated into *Nicotiana benthamiana* leaves. After injection, samples were incubated in the dark for 12 h, followed by normal cultivation for 2–3 days, and then the fluorescence signals were observed using a confocal laser scanning microscope. The vectors 35S::*VvMYBPA1*-YFPn, 35S::*VvMYBPA2*-YFPn, 35S::*VvMYC2*-YFPc and 35S::*VvWDR1*-YFPn are stored in our laboratory. The Primer sequences used in Table A1.

### 4.7. Transformation of Grape Leaves and Fruits

The full-length sequences of *VvWRKY26*, *VvMYC2*, and *VvWDR1* were double-digested and inserted into the Super1300 vector by T4 ligase to construct the recombinant vectors *VvWRKY26*-OE, *VvMYC2*-OE, and *VvWDR1*-OE. According to the distribution of conserved structures in the CDS regions of *VvWRKY26*, *VvMYBPA1*, *VvMYBPA2*, *VvMYC2*, and *VvWDR1*, the 300 bp sense sequence and antisense sequence of the non-conserved sequence were selected, and these sequences were inserted into the pFGC5941 vector via homologous recombination to create the recombinant vectors *VvWRKY26*-RNAi, *VvMYBPA1*-RNAi, *VvMYBPA2*-RNAi, *VvMYC2*-RNAi, and *VvWDR1*-RNAi.

The empty vector plasmids and recombinant vector plasmids were transformed into Agrobacterium tumefaciens strain GV3101, and the Agrobacterium was resuspended in a resuspension buffer (10 mM MES, 10 mM MgCl_2_, 150 μM As, pH = 5.6) with the concentration adjusted to OD600 = 1.0. The infection solutions of different combinations were mixed in a 1:1 ratio and the grape leaves were infected through vacuum infiltration and the fruits through injection. After the infection, the excess resuspension buffer was removed, the leaves were inserted into conical flasks containing distilled water, and the fruits were placed in Petri dishes with distilled water. After dark incubation for 12 h in a light incubator, followed by alternating light and dark cycles, samples were taken at 4 days and 6 days, and stored at −80 °C for future use. The vectors *VvMYBPA1*-OE, *VvMYBPA2*-OE, and *VvWRKY26*-OE were preserved by our laboratory. The Primer sequences used in Table A1.

### 4.8. Transformation of Grape Callus Tissue

The recombinant vector plasmid *VvWRKY26*-OE, preserved in the laboratory, was transformed into Agrobacterium tumefaciens strain GV3101, resuspended, its concentration was adjusted to OD600 = 0.6, and it was left to stand in the dark for 2–3 h. Callus tissues in good growth condition were selected for infection and then inoculated onto induction medium for 2–3 days. After that, they were placed in co-culture medium containing Acetosyrungone for 2 days and then transferred to co-culture medium containing Cef inhibition medium. After ensuring there was no contamination, they were transferred to selection medium containing hygromycin and cultured in the dark. Samples were taken after 30 days for staining, quickly frozen in liquid nitrogen, and stored at −80 °C for future use.

### 4.9. DMACA Staining and Determination of Total PAs

The infected leaves were soaked in ethanol solution containing 30% glacial acetic acid for 20 h, then rinsed with 75% ethanol for 12 h, rinsed with distilled water 3–5 times, and soaked in 0.6% DMACA solution for 5 min. The staining of the leaves was observed and photographed.

One gram of the sample was taken to be tested (the seeds were removed), ground into powder by liquid nitrogen in a 10 mL centrifuge tube, 5 mL of methanol solution containing 2% hydrochloric acid were added, and the sample was extracted by shaking for 24 h at room temperature in the dark. After this, it was centrifuged at 8500 rpm for 10 min, 50 μL of the supernatant was taken in a 10 mL centrifuge tube, 70% acetone solution containing Vc at 1 g/L to 1 mL was added, and then 3 mL of butanol-hydrochloric acid mixture was added. Then 3 mL of butanol-hydrochloric acid mixture (60/40, *v*/*v*, containing 0.3 g/L ferrous sulfate) was added, and the sample was boil and refluxed for 30 min. The water was cooled quickly and left at room temperature for 10 min, then the absorbance value was measured at 550 nm. The sample was placed unheated at room temperature as a reference, and catechin was used as the standard substance to calculate the content of original anthocyanins based on the standard curve.

### 4.10. Determination of Flavan-3-ol Contents

Referencing the method of Wen Pengfei [33], with slight modifications, 0.3 g of the sample was weighed to be tested, ground into a powder using liquid nitrogen, placed in a 10 mL centrifuge tube, 3 mL of 70% methanol was added, and ultrasonic extraction was performed for 20 min under dark conditions. Subsequently, the sample was centrifuged at 4 °C and 12,000 rpm for 10 min. The supernatant was collected and filtered through a 0.45 μm organic membrane, and after constant temperature rotary evaporation, 1 mL of pure water and 2 mL of ethyl acetate were added for extraction. The upper extraction phase was collected, rotary evaporation was performed, and the sample was extracted with 1 mL of pure methanol. The extract was filtered through a 0.22 μm organic microporous membrane and transferred to a liquid chromatography sample vial for injection.

Chromatographic conditions: Ultimate 3000 HPLC analyzer (Thermo Fisher. Shanghai. China), column: Thermo Syncronis C18 column (250 mm × 4.6 mm, 5 μm), UV detection wavelength at 280 nm. Mobile phase: Phase A is pure methanol; Phase B is 1.3% glacial acetic acid; Phase D is 5% methanol. Ultrasonic degassing: The column temperature was set at 30 °C, the flow rate was 1.0 mL/min, and the sample size was 20 μL.

Elution procedure: 0–5 min, 0–20% A; 5–10 min, 20–25% A; 10–25 min, 25–30% A; 25–30 min, 30–20% A; 30–35 min, 20–0% A.

### 4.11. Yeast One-Hybrid Assay

According to the distribution of cis-acting elements within the promoter sequences of structural genes that interact with transcription factors, the sequences containing the corresponding elements to the promoters of *VvANR*, *VvANS*, *VvLAR1*, and *VvLAR2* were amplified, and four recombinant vectors of pAbAi-*VvLAR1*, pAbAi-*VvLAR2*, pAbAi-*VvANR*, and pAbAi-*VvANS* were obtained by inserting them into the pAbAi vector using enzyme digestion ligation.

The pAbAi recombinant vectors were digested with BstB I (NEB) and then transformed into Y1H Gold yeast cells to serve as bait strains. The prey plasmid pGADT7-*VvWRKY26* was transformed into the bait strain. The empty vector pGADT7 and the mutated cis-element vectors were also transformed into the bait strain as negative controls, and the physical interactions were determined based on the growth of colonies on SD/-Leu medium supplemented with the corresponding AbA concentration, and the colonies were grown at 28 °C for 3–5 days. The construction of the mutated cis-element vectors was completed by Sangon Biotech (Shanghai, China). The vector pGADT7-*VvWRKY26* was stored in our laboratory. The Primer sequences used in Table A1.

### 4.12. Yeast Two-Hybrid Assay

Protein interactions were detected through yeast two-hybrid assay. The *VvWRKY26* gene was separately inserted into pGBKT7 and pGADT7 to construct recombinant vectors, and the pGBKT7-*VvWRKY26*, pGBKT7-53, and pGBKT7 empty vectors were transformed into the yeast strain AH109. Self-activation was tested on the SD/-Leu/-Trp medium, and after co-transforming pGBKT7-*VvWRKY26* with pGADT7-*VvMYBPA1*, and pGADT7-*VvMYBPA2*, pGADT7-*VvMYC2*, and pGADT7-*VvWRKY26* with pGBKT7-*VvWDR1*, interactions were assessed on SD/-Leu/-Trp/-His/-Ade medium and SD/-Leu/-Trp/-His/-Ade/X-α-Gal. Additionally, co-transforming pGADT7-T with pGBKT7-Lam or pGADT7-T with pGBKT7-53 served as negative and positive controls. The vectors pGBKT7-*VvWRKY26*, pGADT7-*VvMYBPA1*, pGADT7-*VvMYBPA2*, pGADT7-*VvMYC2*, and pGBKT7-*VvWDR1* are preserved in our laboratory. The Primer sequences used in Table A1.

### 4.13. Dual-Luciferase Assay for Promoter Activity

The *VvLAR2* and *VvANR* promoter fragments were cloned, and the fragments were cloned into the pGreenII 0800-LUC vector as reporters (pro*VvANR* and pro*VvLAR2*) according to the homologous recombination method. For the luciferase assay, *VvWRKY26*-OE, *VvMYBPA1*-OE, *VvMYBPA2*-OE, *VvMYC2*-OE, and *VvWDR1*-OE were used as effector plasmids to transform the Agrobacterium rhizogenes strain GV3101 containing pSoup-p19. An agrobacterium rhizogene carrying an empty effector was used as a control, and the reporter with the plasmid effector was the empty control effector. Bacterial solutions were mixed in a 1:1 (*v*:*v*) ratio and transiently transformed in 4-week-old tobacco leaves, and the luciferase activity was detected on a multifunctional enzyme marker using a dual luciferase detection kit. The Primer sequences used in Table A1.

### 4.14. Statistical Analyses

All experimental data were obtained from more than three independent biological replicates. The data were sorted and analyzed with WPS Office, Excel, and IBM SPSS Statistics 27. Significance analysis and chart creation were performed using GraphPad Prism 10. Image processing was carried out using Photoshop 2022.

## Data Availability

Data are contained within the article.

## Appendix A

**Table A1:** Primer sequences used in this study.

	Primer Name	Primer Sequence (5′→3′)	Enzyme Sites	Product Length (bp)
Gene cloning	CDS-*VvWRKY26*	F:ATGGAGATTAAAGAGTCTGAGAGGATAG		1434
		R:TCATGGTTTCTCTTTATTCGTATGGGC		
	CDS-*VvMYC2*	F:ATGAAAACTGAAATGGGTATGGGAGGA		1827
		R:TTACCCAACTGATGATGACAAGGGC		
	CDS-*VvWDR1*	F:ATGGAGAGATCAAGCCAAGAATCCCA		1011
		R:CTAAACTTTAAGAAGCTGCAGTTTGTTGG		
qRT-PCR	*VvUBQ* (BN000705)	F:GCTCGCTGTTTTGCAGTTCTAC		151
		R:AACATAGGTGAGGCCGCACTT		
	*VvLAR1* (AJ865336)	F:ACGATGTCCGAACACTGAAC		189
		R:TGAACGCCGCTACTACACTC		
	*VvLAR2* (MW753045.1)	F:TCTCGACATACATGATGATGTG		166
		R:TGCAGTTTCTTTGATTGAGTTC		
	*VvANR* (AB199315)	F:AGAACTACAGGAGTTGGGTGAC		168
		R:CCTTGAATTGCTGGCTTG		
	*VvANS* (EF192468.1)	F:TATGAGGGCAAGTGGGTGAC		238
		R:GAGGTGGGAAGAGTGGTGGC		
	*VvWRKY26*	F:GAACAAGCGAGCCCTCTAAG		230
		R:TTCTGCTATCTGCCCATCCA		
	*VvMYBPA1* (AM259485)	F:GTCTCTCATCGCAGGTAGGC		205
		R:GCTGATCTTGACCCTCTTGC		
	*VvMYBPA2* (EU919682.1)	F:GTGCGATGCTCCAAGGTTAT		105
		R:CAGTGCTGAACTTGAGGGC		
	*VvMYC2* (NM_001281045.2)	F:AGGAATCCGAAGTGACCCG		243
		R:GCCACAAATGCTTCCCAGA		
	*VvWDR1* (XM_010664551.3)	F:GGAACAAGCAGGATGTGAGG		224
		R:ATTGGGTCCAGCAAGCGTAG		
Subcellular localization	SL-*VvWRKY26*	F:CGAGCTCATGGAGATTAAAGAGTCTGAG	Sac I	1431
		R:CGGATCCTGGTTTCTCTTTATTCGTATG	BamH I	
BiFC	BiFC-*VvWRKY26*	F:CGGATCCATGGAGATTAAAGAGTCTGAG	BamH I	1431
		R:GCGTCGACTGGTTTCTCTTTATTCGTAT	Sal I	
Overexpression	OE-*VvMYC2*	F:GCTCTAGAATGAAAACTGAAATGGGT	Xba I	1827
		R:GGGGTACCTTACCCAACTGATGAT	Kpn I	
	OE-*VvWDR1*	F:GCTCTAGAATGGAGAGATCAAGCCA	Xba I	1011
		R:GGGGTACCCTAAACTTTAAGAAGCTG	Kpn I	
	OE-*VvMYC2*	F:GCTCTAGAATGAAAACTGAAATGGGT	Xba I	1827
		R:GGGGTACCTTACCCAACTGATGAT	Kpn I	
	OE-*VvWDR1*	F:GCTCTAGAATGGAGAGATCAAGCCA	Xba I	1011
		R:GGGGTACCCTAAACTTTAAGAAGCTG	Kpn I	
RNAi	+RNAi-*VvWRKY26*	F:CATTACAATTACATTTACAATTAATTTGTATCTGATGGAACTGGTCAAGAT		300
		R:TGTAACATAAGAAATTCTTACACTTCTCACTTTTCCTTCTCTTACTCCTG		
	-RNAi-*VvWRKY26*	F:GGTCAATTTGCAGGTATTTTTCTCACTTTTCCTTCTCTTACTCCTG		300
		R:GCAGGACTCTAGGGACTAGTTTTGTATCTGATGGAACTGGTCAAGAT		
	+RNAi-*VvMYBPA1*	F:GGTCAATTTGCAGGTATTTCCATCTCCCCAAACCGGTTAG		300
		R:GCAGGACTCTAGGGACTAGTAGTTGCAGATACTCTTCATACAGTTTG		
	-RNAi-*VvMYBPA1*	F:GGTCAATTTGCAGGTATTTAGTTGCAGATACTCTTCATACAGTTTGTC		300
		R:GCAGGACTCTAGGGACTAGTCCATCTCCCCAAACCGGTTAGA		
	+RNAi-*VvMYBPA2*	F:CATTACAATTACATTTACAATTACGATTTCGACGAAAACCCGTCTC		300
		R:TGTAACATAAGAAATTCTTACACGCCAGTGTCTTGAGCTCCAAAG		
	-RNAi-*VvMYBPA2*	F:GGTCAATTTGCAGGTATTTGCCAGTGTCTTGAGCTCCAAAG		300
		R:GCAGGACTCTAGGGACTAGTCGATTTCGACGAAAACCCGTCT		
	+RNAi-*VvMYC2*	F:CATTACAATTACATTTACAATTACAATCTGGGGACTGGGGAGC		300
		R:TGTAACATAAGAAATTCTTACACAGCCGAAACTCCTCTCTAACCC		
	-RNAi-*VvMYC2*	F:GGTCAATTTGCAGGTATTTAGCCGAAACTCCTCTCTAACCC		300
		R:GCAGGACTCTAGGGACTAGTCAATCTGGGGACTGGGGAGC		
	+RNAi-*VvWDR1*	F:CATTACAATTACATTTACAATTATGGAGAGATCAAGCCAAGAATCCCA		220
		R:TGTAACATAAGAAATTCTTACACTCGAAGGAGAGGCTGGGGTG		
	-RNAi-*VvWDR1*	F:GGTCAATTTGCAGGTATTTTCGAAGGAGAGGCTGGGGTG		220
		R:GCAGGACTCTAGGGACTAGTTGGAGAGATCAAGCCAAGAATCCCA		
Yeast-one hybridization	Q-Y1H-*VvLAR1*	F:CCAAGCTTTCCGATTGTCTCGG	Hind III	142
		R:GGGGTACCCATATAGGGCTGG	Kpn I	
	Q-Y1H-*VvLAR2*	F:CCAAGCTTTGGGGAAGAAGATACCTAT	Hind III	173
		R:GGGGTACCTCTCGAAAAGATAAATTCA	Kpn I	
	Q-Y1H-*VvANR*	F:CCAAGCTTCCTATATCTCAGCCAC	Hind III	242
		R:GGGGTACCCTATGATTCAACAGATG	Kpn I	
	Q-Y1H-*VvANS*	F:CCAAGCTTGGGTTTATCAGAAATTGAC	Hind III	170
		R:GGGGTACCACACGTTTCAGC	Kpn I	
	AD-*VvWRKY26*	F:CGGAATTCATGGAGATTAAAGAGTCTGA	EcoR I	1434
		R:CGGGATCCTCATGGTTTCTCTTTAT	BamH I	
Dual luciferase assay	pro*VvANR*	F:**ACTCACTATAGGGCGAATTGGGTACC** TAGATTTGGATGACTATTGG		1605
		R:**GCGGCCGCTCTAGAACTAGTGGATCC** ATGCCCTCACTTCCAAATTC		
	Pro*VvLAR2*	F:**ACTCACTATAGGGCGAATTG** GATGATAAGATTCAAGTGTCAAGTGC		1770
		R:**GCGGCCGCTCTAGAACTAGT** ATCGGTGTTAATTTTCTGAGAAAGAAG		

Note: The underlined letters are the enzyme sites; bolded letters are homology arm sequences.
